# Supplementary material for: NINJA trial: should the nail plate be replaced or discarded after nail bed repair in children? Protocol for a multicentre randomised control trial
Source: BMJ Open. 2019 Dec 4;9(12):e031552. doi: 10.1136/bmjopen-2019-031552 (PMC6924816; doi:10.1136/bmjopen-2019-031552)
Supplement: Author's manuscript [file bmjopen-2019-031552.draft_revisions.pdf]

BMJ Open is committed to open peer review. As part of this commitment we make the peer review history of every article we publish publicly available.

When an article is published we post the peer reviewers' comments and the authors' responses online. We also post the versions of the paper that were used during peer review. These are the versions that the peer review comments apply to.

The versions of the paper that follow are the versions that were submitted during the peer review process. They are not the versions of record or the final published versions. They should not be cited or distributed as the published version of this manuscript.

BMJ Open is an open access journal and the full, final, typeset and author-corrected version of record of the manuscript is available on our site with no access controls, subscription charges or pay-per-view fees (http://bmjopen.bmj.com).

If you have any questions on BMJ Open's open peer review process please email info.bmjopen@bmj.com

BMJ Open

The NINJA Trial - should the nail plate be replaced or discarded after nail bed repair in children? Protocol for a multi-centre randomised control trial

Journal:	*BMJ Open*
Manuscript ID	bmjopen-2019-031552
Article Type:	Protocol
Date Submitted by the Author:	08-May-2019
Complete List of Authors:	Jain, Abhilash; University of Oxford Nuffield Department of Orthopaedics Rheumatology and Musculoskeletal Sciences, Plastic Surgery; Imperial College Healthcare NHS Trust, Department of Plastic and Reconstructive Surgery<br>Jones, Amy; University of Oxford Nuffield Department of Orthopaedics Rheumatology and Musculoskeletal Sciences<br>Gardiner, Matthew; University of Oxford Nuffield Department of Orthopaedics Rheumatology and Musculoskeletal Sciences, ; Frimley Health NHS Foundation Trust, Plastic Surgery<br>Cooper, Cushlar; University of Oxford Nuffield Department of Orthopaedics Rheumatology and Musculoskeletal Sciences<br>Sierakowski, Adam; Mid Essex Hospital Services NHS Trust, St Andrew's Centre for Plastic Surgery and Burns<br>Dritsaki, Melina; University of Oxford Nuffield Department of Orthopaedics Rheumatology and Musculoskeletal Sciences<br>Png, May ee; University of Oxford, NDORMS<br>Stokes, Jamie; University of Oxford Nuffield Department of Orthopaedics Rheumatology and Musculoskeletal Sciences<br>Shirkey, Beverly; University of Oxford Nuffield Department of Orthopaedics Rheumatology and Musculoskeletal Sciences<br>Cook, Jonathan; University of Oxford Nuffield Department of Orthopaedics Rheumatology and Musculoskeletal Sciences<br>Beard, David; University of Oxford Nuffield Department of Orthopaedics Rheumatology and Musculoskeletal Sciences<br>Greig, Aina; Saint Thomas' Hospital
Keywords:	nail bed, finger, randomised controlled trial, paediatric

SCHOLARONE™
Manuscripts

The NINJA Trial - should the nail plate be replaced or discarded after nail bed repair in children? Protocol for a multi-centre randomised control trial.

Abhilash Jain1,2
Abhilash.jain@kennedy.ox.ac.uk

Amy Jones1
Amy.jones@ndorms.ox.ac.uk

Matthew D. Gardiner1,3
matthew.gardiner@kennedy.ox.ac.uk

Cushla Cooper1
Cushla.cooper@ndorms.ox.ac.uk

Adam Sierakowski4
adam.sierakowski@meht.nhs.uk

Melina Dritsaki1
Melina.dritsaki@ndorms.ox.ac.uk

May Ee Png1
may.png@ndorms.ox.ac.uk

Jamie Stokes1
jamie.stokes@ndorms.ox.ac.uk

Beverly Shirkey1
beverly.shirkey@ndorms.ox.ac.uk

Jonathan Cook1
Jonathan.cook@ndorms.ox.ac.uk

David Beard1
David.beard@ndorms.ox.ac.uk

Aina V.H. Greig5
aina.greig@mac.com

Author affiliations

1. Nuffield Department of Orthopaedics, Rheumatology and Musculoskeletal Sciences, University of Oxford, Oxford, UK
2. Department of Plastic and Reconstructive Surgery, Imperial College Healthcare NHS Trust, London, UK
3. Department of Plastic and Reconstructive Surgery, Frimley Health NHS Foundation Trust, Slough, UK

For peer review only

4. St Andrew's Centre for Plastic Surgery and Burns, Mid Essex Hospital Services NHS Trust, Chelmsford, UK
5. Department of Plastic and Reconstructive Surgery, Guy's and St Thomas' NHS Foundation Trust, London, UK

Corresponding author
Matthew D. Gardiner
Nuffield Department of Orthopaedics, Rheumatology and Musculoskeletal Sciences, University of Oxford, Oxford, UK
matthew.gardiner@kennedy.ox.ac.uk
00 44 7870 619 627

For peer review only

Contributors
AJ: first draft of manuscript, developed research question. AJo refined the protocol and edited the manuscript. CC contributed to the protocol design. AS designed the original protocol. MDG edited manuscript, developed the research question. MD: health economic evaluation. MEP: health economic evaluation. JS and BS: sample size and statistical analysis. JAC: sample size and statistical analysis. DB: developed the research question. AVHG: developed the research question. All authors reviewed and agreed the final manuscript

Funding
AJ, MDG, CC, AS, JC, DB, AVHG obtained grant funding for this project. This project was funded by the UK National Institute for Health Research (NIHR) Research for Patient Benefit (RfPB) grant (PB-PG-1215-20041) and was supported by the NIHR Biomedical Research Centre.

Competing interests
None to declare.

Patient consent for publication
Not required.

Ethics approval
The National Research Ethic Committee approved this study on 2nd February 2018 (18/SC/0024).

Keywords
Nail bed, finger, paediatric, randomised clinical trial

Word count 3752

For peer review only

ABSTRACT

Introduction

Trauma to the nail bed is the commonest surgically treated paediatric hand injury. The majority of surgeons replace the nail plate after repairing the nail bed despite a lack of evidence to do so. Replacing the nail plate may be associated with increased post-operative infection. We will look at the impact of replacing or discarding the nail plate on infection, cosmetic appearance, pain, and subsequent healthcare use. The Nail bed INJury Analysis trial (NINJA) aims to answer the question of whether the nail plate should be replaced or discarded after nail bed repair in children

Methods and Analysis

A two-arm parallel group open multicentre randomized control trial of replacing the nail plate or not, as part of a nail bed repair, will be undertaken in children presenting within 48 hours of a nail bed injury requiring surgical repair. The co-primary outcomes are cosmetic appearance summary score and surgical site infection. Secondary outcomes are EuroQol EQ-5D-(Y); the level of pain experienced at first dressing change; child/parent satisfaction with nail healing and healthcare resource utilization. We will recruit a minimum of 416 patients (208 in each group) over 3 years. Children and their parents/carers will be reviewed in clinic 7-10 days after their operation and will be assessed for infection or other problems. The children and parents/carers will also be asked to complete a questionnaire and send in photos of their fingernail at 4 months post surgery.

Ethics and dissemination

For peer review only

The National Research Ethic Committee approved this study on 2nd February 2018 (18/SC/0024). A manuscript to a peer-reviewed journal will be submitted on completion of the trial as per National Institute for Health Research publication policy. The results of this trial will substantially inform clinical practice and provide evidence on whether the practice of replacing the nail should continue.

Trial registration number ISRCTN44551796, protocol v1.0 08-05-2019

Strengths and limitations of this study

- Pragmatic study design to ensure generalisability.
- First randomised trial to use the Reconstructive Surgery Trials Network
- A health economic evaluation, as well as the clinical assessment will be performed.
- It will not be possible to get patient reported outcomes from all participants owing to their young age.

For peer review only

INTRODUCTION

Nail bed trauma is the most common surgically treated paediatric hand injury and accounts for 10,000 operations annually in the United Kingdom (UK) (1). Surgery involves removing the nail plate (fingernail) and repairing the underlying nail bed laceration with sutures. Once the nail bed has been repaired 96% of surgeons in the UK replace the nail plate (1). The replaced nail plate is eventually pushed out as a new nail grows. It is believed that the replaced nail plate acts as a splint to hold open the nail fold and protect the repair. However, a recent retrospective study of nail bed repairs in children reported a higher infection rate in nail replaced (7.8%, 4 of 51) versus nail discarded groups (0%, 0 of 60) (6). There were also significantly more hospital visits and a longer overall follow up period needed in the nail replaced group compared to the nail discarded group. The suggestion is that the replaced nail acts as a foreign body, which increases the infection risk and wound problems.

A recent Cochrane review found no randomised trials and concluded there was a lack of evidence to inform all key treatment decisions in the management of fingertip entrapment injuries in children (2). Our patient/parent survey identified normal nail re-growth and long-term cosmetic appearance, along with infection risk as the most common concerns following surgery (1). In 2015, we performed a pilot study (NINJA-P) to inform the design and conduct of a definitive trial comparing replacing or discarding the nail after nail bed repair (3). NINJA-P recruited 60 participants at four hand surgery centres over four months. Participants completed follow-up to 4 months. This successful pilot enabled us to demonstrate the viability of a large randomised trial in an area where such trials are rare. It has also enabled us to refine the main trial design including

For peer review only

optimising timing and mode of follow-up as well as providing data, which informed the sample size calculation.

The Nail bed INJury Analysis (NINJA) trial seeks to answer the question “should the nail plate be replaced or discarded after nail bed repair in children, as evaluated by overall infective complications and appearance of the nail (co-primary outcome measures)?” This will help determine whether the simple act of discarding the nail improves the appearance, reduces infection rates and reduces hospital attendances for thousands of children undergoing this operation every year.

Good clinical practice

The NINJA RCT will be carried out in accordance with Medical Research Council Good Clinical Practice and applicable UK legislation whilst following the protocol (V1.0 9 August 2016).

Consolidated standards of reporting trials

The trial will be reported in line with the Consolidated Standards of Reporting Trials statement using the non-pharmacological treatment interventions extension.

Objectives (Table 1)

Primary objectives

To assess the effects of replacing or discarding the fingernail in children undergoing nail bed repair by comparing the risk of infection and cosmetic appearance at 4 months.

Secondary objectives

For peer review only

a) To assess whether there is a difference in participant/parent and guardian reported health-related quality of life according to whether the nail is replaced or discarded.

b) To assess whether there is a difference in participant/parent and guardian reported pain experienced between replacing and discarding the nail at first dressing change.

c) To conduct a parallel within-trial economic analysis to assess the cost-effectiveness of replacing versus discarding the nail.

d) To assess if any infection has occurred within the last 4 months (in addition to early infection with the first 7-10 days).

e) To assess participant/parent satisfaction with nail healing at 4 months.

METHODOLOGY

NINJA is a multicentre, pragmatic 2-arm parallel group superiority randomised controlled trial. Four hundred and sixteen patients will be recruited from up to 30 National Health Service (NHS) hand surgery units across the UK over 18 months (July 2018-December 2019). Participants will be randomised to either have the nail plate replaced or discarded following repair of the nailbed injury. They will be followed by the local clinics at the first routine clinic appointment (between 7-10 days post operation) and will report additional treatments received in the following 4 months. Participants and parents (or guardian) will complete questionnaires at the clinic appointment and report (parent or guardian) questionnaires at 7 days and at 4 months via electronic post, providing photos of the injured and matched opposite finger. If problems are reported via the parent questionnaire, the clinics will be queried for need of

For peer review only

reporting of additional treatment. The trial will run for 3 years. A flowchart depicting the trial process is shown in Figure 1.

Outcome measures

Primary outcome measures

Surgical site infection at 7-10 days

The presence of a surgical site infection (SSI) at 7-10 days post-surgery will be collected. The principal means of data collection will be via a clinical research nurse or attending surgeon assessment of the child's fingertip for absence or presence of infection at the surgical site at the clinical visit. Where appropriate other data sources (e.g. 4 months parent/guardian questionnaire) will be used to supplement this for occurrence of a SSI within the relevant timeframe.

Cosmetic appearance of the nail

The cosmetic appearance of the fingernails will be assessed via a modified version of the Zook score at four months post-randomisation. The modified Zook Score will be a sum of the five components, nail shape, nail adherence, eponychium, nail surface and nail plate split. Each component will be scored as a one if it is deemed to be same as the opposite finger or not having the defect, and zero if the fingernail is deemed to be worse than the opposite finger or if the defect is present. The best score will be a five, and the worst possible score is zero.

The assessors will be made up of surgical trainees, specialist registrars and hand physiotherapists, who will review the photographs submitted at the 4 month follow up time point. The assessors will be blinded to the intervention the participant received, although they may have been involved in the trial at a

For peer review only

participating site (i.e. recruitment, surgery or follow up). Assessors will complete training on the modified Zook Assessment. The first batch of approximately 50 photographs will be assessed for quality control purposes, and if needed, modification to assessment training, instruction to parents, and the modified Zook Assessment may be necessary. If so, the first batch of photographs will be reassessed to new standards. The appearance of the nail will be recorded on the CRF according to the rating system devised by Zook et al and modified for the purposes of this trial (4).

Secondary outcome measures

Health-related quality of life

The EQ-5D-Y is a validated, child-friendly, health-related quality of life questionnaire consisting of five domains related to daily activities with 3-level answer possibilities. This will be completed by the patient (or via parent/guardian proxy depending on the child's age) at baseline, 7-10 days, and 4 months post-randomisation.

Pain at dressing change

The level of pain experienced by the child at their first dressing change (7-10 days) will be assessed using a modified Wong Baker Scale (3 point Likert scale for children). This will be completed by the patient, or a parent/guardian proxy.

Cost-effectiveness

A health resource use questionnaire will be completed by the parent/guardian at 7-10 days and 4 months post-randomisation. This will collect information on hospital visits, dressing and antibiotic use and in some cases hospital readmission and repeat surgery.

For peer review only

Surgical site infection by 4 months

The presence of a SSI by 4 months post-randomisation will be assessed. In addition to the clinical assessment at 7-10 days, the patient's parent/guardian will be asked if the patient experienced any problems post-surgery. This will then be referred back to sites, where appropriate, to obtain confirmation from clinical notes. This will capture any surgical site infections which occur after the usual expected timeframe in which infections would normally present.

Participant/parent satisfaction with nail healing

A modified Wong Baker scale (3-point Likert scale for children) will be used to measure patient reported satisfaction with the healing of the nail at 4 months post-randomisation. If the child cannot complete this score, a Visual Analogue Scale (VAS) in the form of a measured line with a continuous scale (from 0 to 100) anchored by two verbal descriptors for each extreme symptom will be used as a patient proxy for measuring satisfaction with nail healing.

Study population

Inclusion criteria

- Male or female, aged below 16 years old at the time of presentation to the participating hospital.
- Nail bed injury occurring within 48 hours of presentation at trial centre believed to require surgical repair by the surgical team. This includes sharp lacerations, stellate lacerations, crush and avulsion injuries of the nail bed, injuries involving the sterile and/or germinal matrix, nail bed injuries with an associated pulp laceration and/or with an associated 'tuft' fracture of the distal phalanx.

For peer review only

- Patients whose parent or legal guardian consent to their inclusion in the trial and are willing to complete follow up, including photographs.
- Sufficient understanding of the child and parent/guardian participant information sheets as deemed by recruiting team at local sites.
- Single digit nail bed injury.

Exclusion criteria

The participant will not enter the trial if any of the following apply.

- Patients present with an infected nail bed injury.
- Patients have an underlying nail disease or deformity in the injured or contralateral finger prior to the injury.
- Patients have an associated distal phalanx fracture requiring fixation with a Kirschner wire.
- Patients with an amputation of the distal fingertip including all or part of the nail bed.
- Patients with loss of part or all of the nail bed, requiring a nail bed graft or flap reconstruction.
- Previous NINJA trial participants.
- Patients with nail bed injuries to more than one digit.

Recruitment and consent

Trial participants will be prospectively recruited from the participating hospitals. Initial assessment will take place in the Accident and Emergency/Minor Injuries department or paediatric ward. The clinical team will identify any potential participants and refer on to the research team for further information. The

For peer review only

research team will obtain informed consent. Screening logs will be maintained at each site. Reasons for non-participation and/or ineligibility will be documented.

Parents/guardians will be given an information sheet and have the trial explained to them by the researcher. Children will also be provided with age appropriate information in order to include them in the consent process. Consent for medical photography will be included as part of the consent process, and agreement to return follow up questionnaires and submit a photograph at 4 months post-surgery will be part of the inclusion criteria.

Data collection

The baseline assessment will be on the day of the operation, before randomisation but after consent to participation. Participant demographics will be recorded on the Case Report Form (CRF) when the assessing surgeon on admission in the Emergency Department or the paediatric ward sees the participant. Follow up assessments will involve a clinical appointment between 7 and 10 days post operation and a participant reported questionnaire, sent via text, email or post, at 7-10 days post operation and 4 months (Table 1 and Figure 1).

Randomisation and blinding

A web-based randomisation system will be provided by the Oxford Clinical Trials Research Unit (OCTRU). The allocations will be computer generated with a 1:1 ratio and stratified by site using random permuted blocks of varying size within stratum. Randomisation will take place when the participant is in the

For peer review only

anaesthetic room just prior to surgery, or as close to the surgery time as possible by a good clinical practice (GCP) trained member of the team.

This is an open trial, since those delivering the care will not be blinded to the intervention the participant has been allocated to. This is because a replaced nail can take several weeks to loosen and fall off once a new nail has grown out and therefore the treatment received will be obvious within this timeframe. Therefore, the assessment of the photographs for cosmetic appearance at 4 months will be done by independent assessors who can at that time point be blinded.

Operative assessment

At the time of surgery, the operating surgeon will classify the nail bed injury according to the system used and tested in the pilot (3).

Interventions

Nail bed repair

In both groups (nail plate replaced or nail plate discarded), the nail bed repair will be performed using 6/0 or 7/0 interrupted Vicryl Rapide (Johnson and Johnson Medical Ltd, Livingston, West Lothian, UK) or equivalent sutures. This is a pragmatic trial. The following decisions will be left to the discretion of the surgical team responsible for the participant, but recorded on the CRF:

- The type of anaesthetic used (general anaesthetic, local anaesthetic, or both)
- Perioperative antibiotics given, if any
- Type and duration of tourniquet used

For peer review only

- Type of surgical preparation solution and wash used
- Type of dressing applied

If the surgeon has to perform a procedure(s), which was part of the exclusion criteria, this will be recorded on the CRF. This is an extremely unusual event as the vast majority of these procedures (e.g. fracture fixation with a Kirschner wire, need for a composite graft or nail bed graft) are predictable pre-operatively. These participants will be analysed within the intention to treat analysis of the trial. In both groups the fingertip will be dressed with a non-adherent dressing. The operating surgeon will add to the CRF the following data: the type of nail bed injury, whether the nail plate was replaced or discarded, whether a nail substitute was used, what, if any, antibiotics were given perioperatively and what postoperative antibiotic regime is planned.

Nail plate replaced

In the nail plate replaced group, the nail plate will be secured using a figure-of-eight suture. If the nail plate cannot be replaced in a participant randomised to this group, for example if it is too badly damaged, a nail substitute of the operating surgeon's choice will be used and recorded on the CRF.

Nail plate discarded

In the nail plate discarded group after the nail bed repair, the nail will not be replaced. It will be discarded appropriately instead. The washout, debridement, and suturing procedures will be the same as described for the first group.

Safety reporting

For peer review only

Data on complications will be recorded and their severity and frequency will be assessed. Standard HRA safety reporting measures will be adhered to. The OCTRU conducted a risk assessment prior to the trial starting. Issues raised have been addressed within the final protocol and procedures have been planned to monitor the on-going risks of the trial. A risk proportionate approach will be utilised within this trial. Central monitoring of trial procedures will be imbedded into the trial conduct and management, including instituting a trial steering committee (TSC) and data monitoring committee (DMC). The TSC and DMC will agree their respective terms of reference. No formal statistical interim safety analysis has been planned for in the design, or are anticipated given the nature of the trial. The trial may be monitored, or audited in accordance with the current approved protocol, GCP, relevant regulations and standard operating procedures. The trial will be subject to audit according to OCTRU's Audit Programme.

End of trial

The end of trial is the date of the last follow up of the last participant.

Analysis

Statistical analysis

Principal analyses will be on an "as randomised" basis retaining participants in their randomised allocation groups irrespective of compliance to the allocation. A two sided 5% significance level will be adopted with associated 95% confidence intervals (CIs) whenever possible using appropriate summary measures (e.g. number of events and percentage for binary measures). The

For peer review only

principal analyses will also be carried out on a complete case basis with sensitivity to missing data explored for the primary outcomes.

The number of participants

Sample size calculations are based on the co-primary outcomes of surgical site infection and cosmetic appearance at 4 months, measured via a modified Oxford cosmetic nail score based upon the Zook classification scale (4) – a 0-5 ordinal summary score reflecting optimal or suboptimal appearance across the five classification domains. Pilot data from our NINJA-P trial (3) showed a substantial proportion of participants did not have nails with optimal appearance (approximately 35% had two or more suboptimal aspects of appearance, i.e. score of three or less). Based upon a clinically relevant difference of 15% more achieving the optimal appearance score of 5 (from 35 to 50% with a corresponding shift in the other score values) and using a two-sided significance level of 0.05, 332 (166 per group) are required to obtain 90% power based upon a Mann-Whitney U test. After allowance for 20% missing data, a total of 416 participants (208 in each group) are required. This calculation was carried out using an extended version of the Excel spreadsheet provided by Walters (5) to allow for a six point ordinal outcome. Based upon a lower overall level and a smaller difference in the proportion with a surgical site infection than the one observed in the Miranda (6) observational study (8 vs 1%), this sample size is also sufficient for 90% power at the two-sided 5% significance level. This latter calculation was carried out in Stata 14 using the power twoprop command.

For peer review only

Analysis of outcome measures

As multiple assessors will be reviewing each photograph using the modified Zook cosmetic appearance score, the median of the assessors' total scores will be used as the rating for each photo to account for any variability in scores. These will then be analysed using a Mann-Whitney U test (with a 95% CI for the median also calculated). A secondary more complex ordinal regression model will also be used to estimate the difference across the ordinal scale and allow subgroup analyses.

Surgical site infection will be compared using logistic regression adjusted for site. Pre-specified subgroup analysis will be carried out according to preoperative antibiotic use using a treatment-by-subgroup interaction extending the aforementioned regression models for the co-primary outcomes. Secondary outcomes will be analysed using generalised linear models as appropriate. Further details of the planned statistical analyses will be specified in a Statistical Analysis Plan, which will be finalised prior to the un-blinding of data to NINJA investigators. Available data will be used up to the point of withdrawal whenever possible.

Economic analysis

A within-trial cost-utility analysis comparing nail replacement with nail discarding will be conducted from the UK NHS and Personal Social Services perspective over 4 months in the base case (or primary) analysis (7).

Resource use for the surgery will be recorded by the research team in the CRF while data for the economic evaluation will be collected from the trial questionnaires given to participants at 7-10 days and 4 months after

For peer review only

randomisation. Unit cost of this resource use will be sourced from the latest NHS Supply Chain Catalogue, NHS Reference Cost and British National Formulary. Where appropriate, the cost of health resource use per patient will be computed by multiplying the frequency of health resource utilisation with the unit cost of each resource item.

Health-related quality of life (HRQoL) will be estimated using the EQ-5D-Y questionnaire at baseline, 7-10 days and 4 months. The EQ-5D-Y user guide instructions will be followed as much as possible so that children are given age-appropriate questionnaires to answer (8).

A cost-utility analysis (excluding the participants below the age of 2) will present outputs of the analyses in terms of incremental cost-effectiveness ratio (ICER) where the NICE cost-effectiveness threshold of £20,000-£30,000 per additional QALY will be applied. Given the methodological limitations surrounding preference-based outcomes measurement in young children, a cost-effectiveness analysis will also be conducted (for the entire sample) where outputs will be expressed in terms of incremental cost per surgical site infections prevented.

If data are missing at random, multiple imputation analysis will be performed to avoid bias associated with the complete case analysis. We assume no outcome differences in terms of QoL, pain and complications beyond the trial period, therefore no longer-time perspective (beyond 4 months) will be considered.

Sensitivity analysis such as extending the study perspective to societal perspective and assessing the impact of missing data on the ICERs will be performed. In order to assess sampling uncertainty on the ICERs and varying willingness-to-pay levels for an additional QALY, probabilistic sensitivity

For peer review only

analysis (PSA) will be performed. Results from the PSA will be presented in cost-effectiveness acceptability curves, which will be generated via non-parametric bootstrapping.

Patient and public involvement

To inform study design 30 parents of children with nail bed injuries were surveyed. The survey identified normal regrowth of the nail, infection and long-term appearance as the most common parental concerns following nail bed surgery (1). Subsequently a focus group and youth group refined follow-up methods, types of study material, as well as which outcomes were important. To ensure on-going patient and public involvement, a patient/carer representative is actively involved in general trial management. In addition, further independent patient/carer representatives are members of the steering committee.

Ethics and dissemination

This trial is conducted in accordance with the principles of the Declaration of Helsinki, with relevant regulations and with Good Clinical Practice. It has been approved by the South Central Research Ethics Committee (Berkshire-B, 20/02/2018, ref: 18/SC/0024). The participants in this trial are children and consent for them to take part will need to be obtained from their parent or legal guardian by a GCP trained research team member. If a child wishes not to take part in the trial, this will be respected. Personal information will be handled confidentially in line with GDPR regulations. Any publication arising out of the trial will follow the NIHR publication policy.

For peer review only

REFERENCES

1. Sierakowski A, Gardiner MD, Jain A, Greig AV, Nail bed INJury Analysis (NINJA) Collaborative Group. Surgical treatment of paediatric nail bed injuries in the United Kingdom: Surgeon and patient priorities for future research. J Plast Reconstr Aesthet Surg. 2016 Feb;69(2):286–8.

2. Capstick R, Giele H. Interventions for treating fingertip entrapment injuries in children. Capstick R, editor. Cochrane database of systematic reviews (Online). Chichester, UK: John Wiley & Sons, Ltd; 2014 Apr 30;(4):CD009808.

3. Greig A, Gardiner MD, Sierakowski A, Zweifel CJ, Pinder RM, Furniss D, et al. Randomized feasibility trial of replacing or discarding the nail plate after nail-bed repair in children. Br J Surg. 2017 Nov;104(12):1634–9.

4. Zook EG, Guy RJ, Russell RC. A study of nail bed injuries: causes, treatment, and prognosis. YJHSU. 1984 Mar;9(2):247–52.

5. Walters SJ. Sample size and power estimation for studies with health related quality of life outcomes: a comparison of four methods using the SF-36. Health Qual Life Outcomes. 2004 May 25;2:26.

6. Miranda BH, Vokshi I, Milroy CJ. Pediatric Nailbed Repair Study. Plast Reconstr Surg. 2012 Feb;129(2):394e–396e.

7. National Institute for Health and Care Excellence. Guide to the Methods of Technology Appraisal 2013. London: National Institute for Health and Care Excellence (NICE); 2013.

For peer review only

8. Van Reenen M, Janssen B, Oppe M, Group SKRE, 2014. EQ-5D-Y user guide: basic information on how to use the EQ-5D-Y instrument.

9. Chen G, Ratcliffe J. A Review of the Development and Application of Generic Multi-Attribute Utility Instruments for Paediatric Populations. Pharmacoeconomics. 2015 Oct;33(10):1013–28.

For peer review only

Table 1. Objectives and outcome measures

Objectives	Outcome Measures	Timepoint(s) of evaluation of this outcome measure (if applicable)
**Primary Objectives**<br>To assess the effects of replacing or discarding the fingernail by comparing the risk of infection and cosmetic appearance.	• Incidence of surgical site infection (clinical assessment at 7-10 days and participant or parent/guardian reported with clinical notes at 4 months).<br>• Modified Zook Score assessing nail appearance at 4 months, considering 5 domains (shape, adherence, eponychium, surface quality and presence of split).	• 7-10 days<br>• At 4 months
**Secondary Objectives**<br>To assess whether there is a difference in participant/parent & guardian reported health-related quality of life according to whether the nail is replaced or discarded.	• EuroQol EQ-5D-Y, and proxy completed by the child/parent or guardian according to the age of the participant	• Baseline, 7-10 days and 4 months
To assess whether there is a difference in participant or parent/guardian -reported pain experienced between replacing and discarding the nail.	• The level of pain experienced by the child at their first dressing change assessed by the child or parent/guardian (3 point Likert scale for children [modified Wong Baker scale])	• 7-10 days
To conduct a parallel within-trial economic analysis to assess the cost effectiveness (including resource use) of replacing versus discarding the nail	• Healthcare resource utilisation such as increased hospital visits, dressing and antibiotic use and in some cases hospital readmission and repeat surgery.	• 7-10 days and 4 months
To assess if any surgical site infection has occurred within the last 4 months.	• Participant or parent/guardian reported incidence of infection with clinical notes confirmation.	• At 4 months
To assess participant/parent satisfaction with nail healing	• Child or parent/guardian satisfaction with nail healing (3 point Likert scale for the children [modified Wong Baker scale] and a VAS score for the parents/guardians).	• 4 months

Figure 1. Trial flow chart

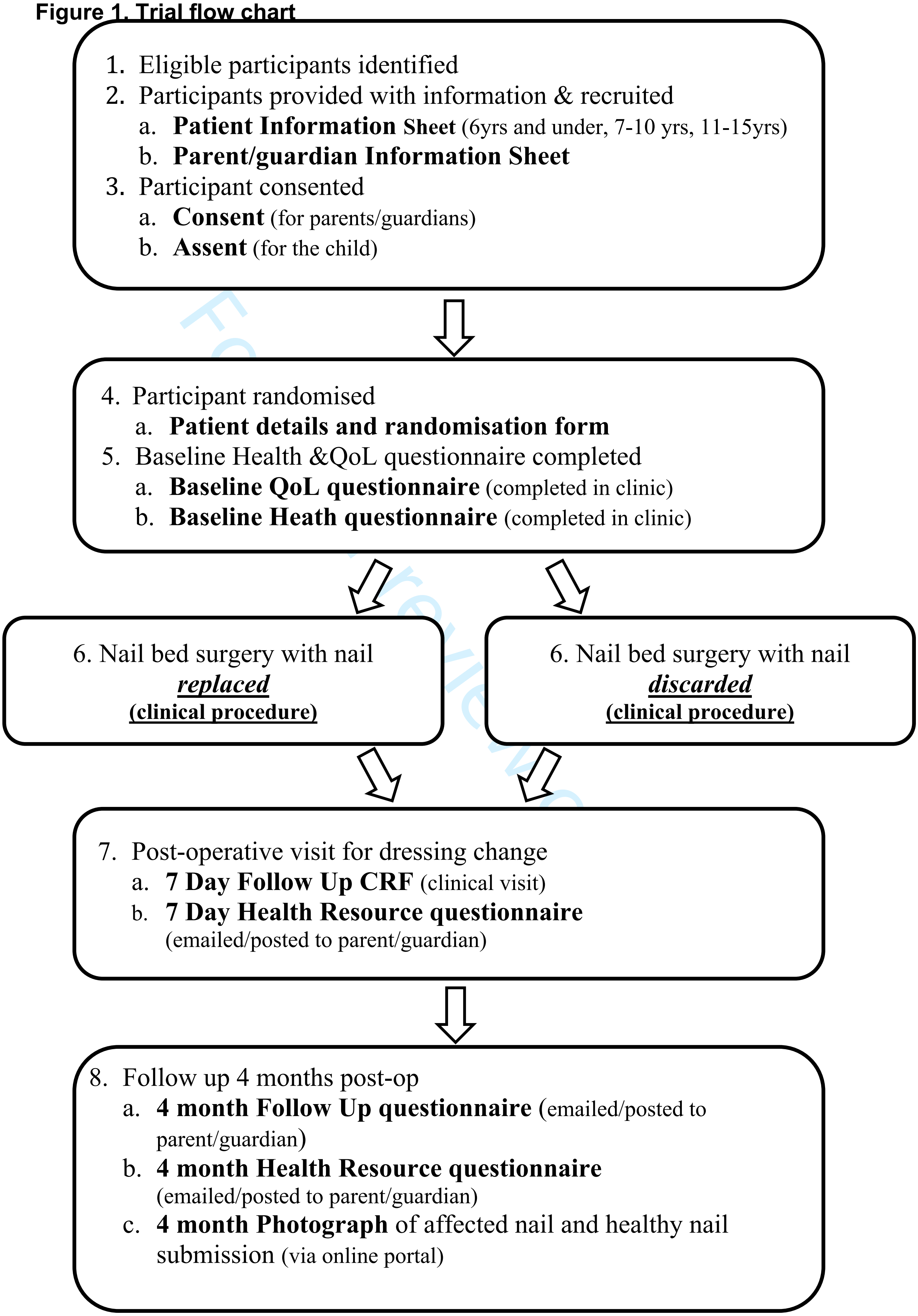

Reporting checklist for protocol of a clinical trial.

Based on the SPIRIT guidelines.

Instructions to authors

Complete this checklist by entering the page numbers from your manuscript where readers will find each of the items listed below.

Your article may not currently address all the items on the checklist. Please modify your text to include the missing information. If you are certain that an item does not apply, please write "n/a" and provide a short explanation.

Upload your completed checklist as an extra file when you submit to a journal.

In your methods section, say that you used the SPIRITreporting guidelines, and cite them as:

Chan A-W, Tetzlaff JM, Altman DG, Laupacis A, Gøtzsche PC, Krleža-Jerić K, Hróbjartsson A, Mann H, Dickersin K, Berlin J, Doré C, Parulekar W, Summerskill W, Groves T, Schulz K, Sox H, Rockhold FW, Rennie D, Moher D. SPIRIT 2013 Statement: Defining standard protocol items for clinical trials. Ann Intern Med. 2013;158(3):200-207

		Reporting Item	Page Number
**Administrative information**			
Title	#1	Descriptive title identifying the study design, population, interventions, and, if applicable, trial acronym	1
Trial registration	#2a	Trial identifier and registry name. If not yet registered, name of intended registry	5
Trial registration: data set	#2b	All items from the World Health Organization Trial Registration Data Set	5
Protocol version	#3	Date and version identifier	5
Funding	#4	Sources and types of financial, material, and other support	3
Roles and responsibilities: contributorship	#5a	Names, affiliations, and roles of protocol contributors	2

For peer review only

Roles and responsibilities: sponsor contact information	#5b	Name and contact information for the trial sponsor	2
Roles and responsibilities: sponsor and funder	#5c	Role of study sponsor and funders, if any, in study design; collection, management, analysis, and interpretation of data; writing of the report; and the decision to submit the report for publication, including whether they will have ultimate authority over any of these activities	2
Roles and responsibilities: committees	#5d	Composition, roles, and responsibilities of the coordinating centre, steering committee, endpoint adjudication committee, data management team, and other individuals or groups overseeing the trial, if applicable (see Item 21a for data monitoring committee)	2
**Introduction**			
Background and rationale	#6a	Description of research question and justification for undertaking the trial, including summary of relevant studies (published and unpublished) examining benefits and harms for each intervention	6
Background and rationale: choice of comparators	#6b	Explanation for choice of comparators	6
Objectives	#7	Specific objectives or hypotheses	7
Trial design	#8	Description of trial design including type of trial (eg, parallel group, crossover, factorial, single group), allocation ratio, and framework (eg, superiority, equivalence, non-inferiority, exploratory)	8
**Methods: Participants, interventions, and outcomes**			
Study setting	#9	Description of study settings (eg, community clinic, academic hospital) and list of countries where data will be collected. Reference to where list of study sites can be obtained	8
Eligibility criteria	#10	Inclusion and exclusion criteria for participants. If applicable, eligibility criteria for study centres and individuals who will	11

For peer review only

		perform the interventions (eg, surgeons, psychotherapists)	
Interventions: description	#11a	Interventions for each group with sufficient detail to allow replication, including how and when they will be administered	8
Interventions: modifications	#11b	Criteria for discontinuing or modifying allocated interventions for a given trial participant (eg, drug dose change in response to harms, participant request, or improving / worsening disease)	8
Interventions: adherance	#11c	Strategies to improve adherence to intervention protocols, and any procedures for monitoring adherence (eg, drug tablet return; laboratory tests)	12
Interventions: concomitant care	#11d	Relevant concomitant care and interventions that are permitted or prohibited during the trial	12
Outcomes	#12	Primary, secondary, and other outcomes, including the specific measurement variable (eg, systolic blood pressure), analysis metric (eg, change from baseline, final value, time to event), method of aggregation (eg, median, proportion), and time point for each outcome. Explanation of the clinical relevance of chosen efficacy and harm outcomes is strongly recommended	9
Participant timeline	#13	Time schedule of enrolment, interventions (including any run-ins and washouts), assessments, and visits for participants. A schematic diagram is highly recommended (see Figure)	8
Sample size	#14	Estimated number of participants needed to achieve study objectives and how it was determined, including clinical and statistical assumptions supporting any sample size calculations	17
Recruitment	#15	Strategies for achieving adequate participant enrolment to reach target sample size	17
**Methods: Assignment of interventions (for controlled trials)**			
Allocation: sequence generation	#16a	Method of generating the allocation sequence (eg, computer-generated random numbers), and list of any factors for stratification. To reduce predictability of a random sequence, details of any planned restriction (eg, blocking) should be provided in a separate document that is unavailable to those who enrol participants or assign interventions	13

For peer review only

Allocation concealment mechanism	#16b	Mechanism of implementing the allocation sequence (eg, central telephone; sequentially numbered, opaque, sealed envelopes), describing any steps to conceal the sequence until interventions are assigned	13
Allocation: implementation	#16c	Who will generate the allocation sequence, who will enrol participants, and who will assign participants to interventions	13
Blinding (masking)	#17a	Who will be blinded after assignment to interventions (eg, trial participants, care providers, outcome assessors, data analysts), and how	13
Blinding (masking): emergency unblinding	#17b	If blinded, circumstances under which unblinding is permissible, and procedure for revealing a participant's allocated intervention during the trial	13
**Methods: Data collection, management, and analysis**			
Data collection plan	#18a	Plans for assessment and collection of outcome, baseline, and other trial data, including any related processes to promote data quality (eg, duplicate measurements, training of assessors) and a description of study instruments (eg, questionnaires, laboratory tests) along with their reliability and validity, if known. Reference to where data collection forms can be found, if not in the protocol	13
Data collection plan: retention	#18b	Plans to promote participant retention and complete follow-up, including list of any outcome data to be collected for participants who discontinue or deviate from intervention protocols	13
Data management	#19	Plans for data entry, coding, security, and storage, including any related processes to promote data quality (eg, double data entry; range checks for data values). Reference to where details of data management procedures can be found, if not in the protocol	12
Statistics: outcomes	#20a	Statistical methods for analysing primary and secondary outcomes. Reference to where other details of the statistical analysis plan can be found, if not in the protocol	16
Statistics: additional analyses	#20b	Methods for any additional analyses (eg, subgroup and adjusted analyses)	16

For peer review only

Statistics: analysis population and missing data	#20c	Definition of analysis population relating to protocol non-adherence (eg, as randomised analysis), and any statistical methods to handle missing data (eg, multiple imputation)	16
**Methods: Monitoring**			
Data monitoring: formal committee	#21a	Composition of data monitoring committee (DMC); summary of its role and reporting structure; statement of whether it is independent from the sponsor and competing interests; and reference to where further details about its charter can be found, if not in the protocol. Alternatively, an explanation of why a DMC is not needed	15
Data monitoring: interim analysis	#21b	Description of any interim analyses and stopping guidelines, including who will have access to these interim results and make the final decision to terminate the trial	15
Harms	#22	Plans for collecting, assessing, reporting, and managing solicited and spontaneously reported adverse events and other unintended effects of trial interventions or trial conduct	15
Auditing	#23	Frequency and procedures for auditing trial conduct, if any, and whether the process will be independent from investigators and the sponsor	15
**Ethics and dissemination**			
Research ethics approval	#24	Plans for seeking research ethics committee / institutional review board (REC / IRB) approval	3
Protocol amendments	#25	Plans for communicating important protocol modifications (eg, changes to eligibility criteria, outcomes, analyses) to relevant parties (eg, investigators, REC / IRBs, trial participants, trial registries, journals, regulators)	15
Consent or assent	#26a	Who will obtain informed consent or assent from potential trial participants or authorised surrogates, and how (see Item 32)	12
Consent or assent: ancillary studies	#26b	Additional consent provisions for collection and use of participant data and biological specimens in ancillary studies, if applicable	12
Confidentiality	#27	How personal information about potential and enrolled participants will be collected, shared, and maintained in order to protect confidentiality before, during, and after the trial	20

For peer review only

Declaration of interests	#28	Financial and other competing interests for principal investigators for the overall trial and each study site	3
Data access	#29	Statement of who will have access to the final trial dataset, and disclosure of contractual agreements that limit such access for investigators	16
Ancillary and post trial care	#30	Provisions, if any, for ancillary and post-trial care, and for compensation to those who suffer harm from trial participation	n/a
Dissemination policy: trial results	#31a	Plans for investigators and sponsor to communicate trial results to participants, healthcare professionals, the public, and other relevant groups (eg, via publication, reporting in results databases, or other data sharing arrangements), including any publication restrictions	20
Dissemination policy: authorship	#31b	Authorship eligibility guidelines and any intended use of professional writers	20
Dissemination policy: reproducible research	#31c	Plans, if any, for granting public access to the full protocol, participant-level dataset, and statistical code	20
**Appendices**			
Informed consent materials	#32	Model consent form and other related documentation given to participants and authorised surrogates	n/a
Biological specimens	#33	Plans for collection, laboratory evaluation, and storage of biological specimens for genetic or molecular analysis in the current trial and for future use in ancillary studies, if applicable	n/a

The SPIRIT checklist is distributed under the terms of the Creative Commons Attribution License CC-BY-ND 3.0. This checklist was completed on 08. May 2019 using https://www.goodreports.org/, a tool made by the EQUATOR Network in collaboration with Penelope.ai

For peer review only

BMJ Open

The NINJA Trial - should the nail plate be replaced or discarded after nail bed repair in children? Protocol for a multi-centre randomised control trial

Journal:	*BMJ Open*
Manuscript ID	bmjopen-2019-031552.R1
Article Type:	Protocol
Date Submitted by the Author:	20-Aug-2019
Complete List of Authors:	Jain, Abhilash; University of Oxford Nuffield Department of Orthopaedics Rheumatology and Musculoskeletal Sciences, Plastic Surgery; Imperial College Healthcare NHS Trust, Department of Plastic and Reconstructive Surgery<br>Jones, Amy; University of Oxford Nuffield Department of Orthopaedics Rheumatology and Musculoskeletal Sciences<br>Gardiner, Matthew; University of Oxford Nuffield Department of Orthopaedics Rheumatology and Musculoskeletal Sciences, ; Frimley Health NHS Foundation Trust, Plastic Surgery<br>Cooper, Cushlar; University of Oxford Nuffield Department of Orthopaedics Rheumatology and Musculoskeletal Sciences<br>Sierakowski, Adam; Mid Essex Hospital Services NHS Trust, St Andrew's Centre for Plastic Surgery and Burns<br>Dritsaki, Melina; University of Oxford Nuffield Department of Orthopaedics Rheumatology and Musculoskeletal Sciences<br>Png, May ee; University of Oxford, NDORMS<br>Stokes, Jamie; University of Oxford Nuffield Department of Orthopaedics Rheumatology and Musculoskeletal Sciences<br>Shirkey, Beverly; University of Oxford Nuffield Department of Orthopaedics Rheumatology and Musculoskeletal Sciences<br>Cook, Jonathan; University of Oxford Nuffield Department of Orthopaedics Rheumatology and Musculoskeletal Sciences<br>Beard, David; University of Oxford Nuffield Department of Orthopaedics Rheumatology and Musculoskeletal Sciences<br>Greig, Aina; Saint Thomas' Hospital
**Primary Subject Heading**:	Surgery
Secondary Subject Heading:	Paediatrics
Keywords:	nail bed, finger, randomised controlled trial, paediatric

SCHOLARONE™
Manuscripts

The NINJA Trial - should the nail plate be replaced or discarded after nail bed repair in children? Protocol for a multi-centre randomised control trial.

Abhilash Jain1,2
Abhilash.jain@kennedy.ox.ac.uk

Amy Jones1
Amy.jones@ndorms.ox.ac.uk

Matthew D. Gardiner1,3
matthew.gardiner@kennedy.ox.ac.uk

Cushlar Cooper1
Cushla.cooper@ndorms.ox.ac.uk

Adam Sierakowski4
adam.sierakowski@meht.nhs.uk

Melina Dritsaki1
Melina.dritsaki@ndorms.ox.ac.uk

May Ee Png1
may.png@ndorms.ox.ac.uk

Jamie Stokes1
jamie.stokes@ndorms.ox.ac.uk

Beverly Shirkey1
beverly.shirkey@ndorms.ox.ac.uk

Jonathan A. Cook1
Jonathan.cook@ndorms.ox.ac.uk

David Beard1
David.beard@ndorms.ox.ac.uk

Aina V.H. Greig5
aina.greig@gstt.nhs.uk

Author affiliations
1. Nuffield Department of Orthopaedics, Rheumatology and Musculoskeletal Sciences, University of Oxford, Oxford, UK
2. Department of Plastic and Reconstructive Surgery, Imperial College Healthcare NHS Trust, London, UK
3. Department of Plastic and Reconstructive Surgery, Frimley Health NHS Foundation Trust, Slough, UK

For peer review only

For peer review only - http://bmjopen.bmj.com/site/about/guidelines.xhtml

4. St Andrew's Centre for Plastic Surgery and Burns, Mid Essex Hospital Services NHS Trust, Chelmsford, UK
5. Department of Plastic and Reconstructive Surgery, Guy's and St Thomas' NHS Foundation Trust, London, UK

Corresponding author
Matthew D. Gardiner
Nuffield Department of Orthopaedics, Rheumatology and Musculoskeletal Sciences, University of Oxford, Oxford, UK
matthew.gardiner@kennedy.ox.ac.uk
00 44 7870 619 627

For peer review only

For peer review only - http://bmjopen.bmj.com/site/about/guidelines.xhtml

Contributors
AJ: developed research question, contributed to protocol design, first draft of manuscript,. AJo refined the protocol and edited the manuscript. CC contributed to the protocol design. AS and AVHG developed the original research question and protocol, edited manuscript. MDG contributed to research question, protocol design and edited manuscript,. MD: health economic evaluation. MEP: health economic evaluation. JS and BS: sample size and statistical analysis. JAC: sample size and statistical analysis. DB: developed the research question. All authors reviewed and agreed the final manuscript

Funding
AJ, MDG, CC, AS, JC, DB, AVHG obtained grant funding for this project. This project was funded by the UK National Institute for Health Research (NIHR) Research for Patient Benefit (RfPB) grant (PB-PG-1215-20041) and was supported by the NIHR Biomedical Research Centre.

Competing interests
None to declare.

Patient consent for publication
Not required.

Ethics approval
The National Research Ethic Committee approved this study on 4th June 2019 (18/SC/0024).

Keywords
Nail bed, finger, paediatric, randomised clinical trial

Word count 3752

For peer review only

ABSTRACT

Introduction

Trauma to the nail bed is the commonest surgically treated paediatric hand injury. The majority of surgeons replace the nail plate after repairing the nail bed despite a lack of evidence to do so. Replacing the nail plate may be associated with increased post-operative infection. We will investigate the impact of replacing or discarding the nail plate on infection, cosmetic appearance, pain, and subsequent healthcare use. The Nail bed INJury Analysis trial (NINJA) aims to answer the question of whether the nail plate should be replaced or discarded after surgical nail bed repair in children

Methods and Analysis

A two-arm parallel group open multicentre randomized control trial of replacing the nail plate or not, as part of a nail bed repair, will be undertaken in children presenting within 48 hours of a nail bed injury requiring surgical repair. The co-primary outcomes are: cosmetic appearance summary score at a minimum of 4 months and surgical site infection at around 7 days following surgery. Secondary outcomes are EuroQol EQ-5D-(Y); the pain intensity experienced at first dressing change; child/parent satisfaction with nail healing and healthcare resource utilization. We will recruit a minimum of 416 patients (208 in each group) over 3 years. Children and their parents/carers will be reviewed in clinic around 7 days after their operation and will be assessed for surgical site infection or other problems. The children, or depending on age, their parents/carers, will also be asked to complete a questionnaire and send in photos of their fingernail at a minimum of 4 months post surgery to assess cosmetic appearance.

For peer review only - http://bmjopen.bmj.com/site/about/guidelines.xhtml

Ethics and dissemination

The National Research Ethic Committee approved this study on 4th June 2019 (18/SC/0024). A manuscript to a peer-reviewed journal will be submitted on completion of the trial as per National Institute for Health Research publication policy. The results of this trial will substantially inform clinical practice and provide evidence on whether the practice of replacing the nail plate should continue at the time of nail bed repair.

Trial registration number ISRCTN44551796, protocol v3.0 04-06-2019

Strengths and limitations of this study

- Pragmatic study design to ensure generalisability.
- First randomised trial to use the Reconstructive Surgery Trials Network
- A health economic evaluation, as well as the clinical assessment will be performed.
- It will not be possible to get patient reported outcomes from all participants owing to their young age.

For peer review only

INTRODUCTION

Nail bed trauma is the most common surgically treated paediatric hand injury and accounts for 10,000 operations annually in the United Kingdom (UK) (1). Surgery involves removing the nail plate (fingernail) and repairing the underlying nail bed laceration with sutures. Once the nail bed has been repaired 96% of surgeons in the UK replace the nail plate (1). The replaced nail plate is eventually pushed out as a new nail grows. It is believed that the replaced nail plate acts as a splint to hold open the nail fold and protect the repair. However, a recent retrospective study of nail bed repairs in children reported a higher infection rate in the nail replaced (7.8%, 4 of 51) versus nail discarded groups (0%, 0 of 60) (2). There were also significantly more hospital visits and a longer overall follow up period needed in the nail replaced group compared to the nail discarded group. The hypothesis is that the replaced nail plate acts as a foreign body, which increases the infection risk and wound problems.

A recent Cochrane review found no randomised trials and concluded there was a lack of evidence to inform all key treatment decisions in the management of fingertip entrapment injuries in children (3). Our patient/parent survey identified normal nail re-growth and long-term cosmetic appearance, along with infection risk as the most common concerns following surgery (1). In 2015, we performed a pilot study (NINJA-P) to inform the design and conduct of a definitive trial comparing replacing or discarding the nail after nail bed repair (4). NINJA-P recruited 60 participants (age range <1-16 years) at four hand surgery centres over four months. Participants completed follow-up to 4 months. This successful pilot enabled us to demonstrate the viability of a large randomised trial in an area where such trials are rare. It has also enabled us to refine the

For peer review only - http://bmjopen.bmj.com/site/about/guidelines.xhtml

main trial design including optimising timing and mode of follow-up as well as providing data, which informed the sample size calculation.

The Nail bed INJury Analysis (NINJA) trial seeks to answer the question “should the nail plate be replaced intra-operatively or discarded after nail bed repair in children, as evaluated by surgical site infections and appearance of the nail (co-primary outcome measures)?” This will help determine whether the simple act of discarding the nail improves the appearance, reduces infection rates and reduces hospital attendances for thousands of children undergoing this operation every year.

Good clinical practice

The NINJA RCT will be carried out in accordance with Medical Research Council Good Clinical Practice and applicable UK legislation whilst following the protocol (V3.0 4 June 2019).

Consolidated standards of reporting trials

The trial will be reported in line with the Consolidated Standards of Reporting Trials statement using the non-pharmacological treatment interventions extension.

Objectives

Primary objectives

To assess the effects of replacing or discarding the fingernail in children undergoing surgical nail bed repair by comparing the risk of early nail-related surgical site infection and cosmetic appearance at a minimum of 4 months (Table 1).

Secondary objectives

For peer review only - http://bmjopen.bmj.com/site/about/guidelines.xhtml

a) To assess whether there is a difference in participant/parent/guardian reported health-related quality of life according to whether the nail is replaced or discarded.

b) To assess whether there is a difference in participant/parent/guardian reported pain experienced between replacing and discarding the nail at first dressing change.

c) To conduct a parallel within-trial economic analysis to assess the cost-effectiveness of replacing versus discarding the nail.

d) To assess if any late nail-related surgical site infection (e.g. osteomyelitis) has occurred within the last 4 months (in addition to early infection with the first 7 days).

e) To assess participant/parent/guardian satisfaction with nail healing at a minimum of 4 months.

METHODOLOGY

NINJA is a multicentre, pragmatic 2-arm parallel group superiority randomised controlled trial. A minimum of 416 patients will be recruited from up to 30 National Health Service (NHS) hand surgery units across the UK over 18 months (July 2018-December 2019). Participants will be randomised to either have the nail plate replaced or discarded following repair of the nailbed injury. They will be followed by their local clinics at the first routine clinic appointment (around 7 days post operation) and will report additional treatments received in the following 4 months. Participants and parents (or guardian) will complete questionnaires at the clinic appointment and report (parent or guardian) questionnaires at around 7 days and at 4 months via electronic post. Parents

For peer review only - http://bmjopen.bmj.com/site/about/guidelines.xhtml

will provide photos of the injured and matched opposite finger at a minimum of 4 months following surgery. If problems are reported via the parent questionnaire, the clinics will be queried for need of reporting of additional treatment. The trial will run for 3 years. A flowchart depicting the trial process is shown in Figure 1.

Outcome measures

Primary outcome measures

Surgical site infection at 7 days

The presence of a surgical site infection (SSI) at 7 days post-surgery will be collected. The principal means of data collection will be via a clinical research nurse or attending surgeon assessment of the child's fingertip for absence or presence of infection at the surgical site at the clinical visit. It is often difficult to accurately assess infection in very young children and as this is a pragmatic study clinical judgment of infection will be used and is likely to be based on redness, localised pain, presence of pus and fever. Treatment with antibiotics and return to theatre for infective complications will also suggest a diagnosis of infection. Simple inflammation and non-specific pain following this trauma and surgery are not always markers for surgical site infection in this patient population. Where appropriate other data sources (e.g. 4 months parent/guardian questionnaire) will be used to supplement this for occurrence of a SSI within the relevant timeframe.

Cosmetic appearance of the nail

The cosmetic appearance of the fingernails will be assessed using the Oxford Finger Nail Appearance Score at least four months post-randomisation. The

For peer review only - http://bmjopen.bmj.com/site/about/guidelines.xhtml

Score will be a sum of the five components, nail shape, nail adherence, eponychium, nail surface and nail plate split. Each component will be given a score of one if it is deemed to be same as the opposite finger or not having the defect, and a score of zero if the fingernail is deemed to be worse than the opposite finger or if the defect is present. The best total score will be a five, and the worst possible score is zero.

The assessors will be made up of surgical trainees, specialist registrars and hand physiotherapists, who will review the photographs submitted at the minimum 4 months follow up time point. The assessors will be blinded to the intervention the participant received, although they may have been involved in the trial at a participating site (i.e. recruitment, surgery or follow up). Assessors will complete training on the Oxford Finger Nail Appearance Score. The first batch of approximately 50 photographs will be assessed for quality control purposes, and if needed, modification to assessment training, instruction to parents, and the Oxford Finger Nail Appearance Score may be necessary. If so, the first batch of photographs will be reassessed to the new standards. The appearance of the nail will be assessed on the CRF using the Oxford Finger Nail Appearance Score, and the development of this was informed by the Zook nail classification scale (5).

Secondary outcome measures

Health-related quality of life

The EQ-5D-Y is a validated, child-friendly, health-related quality of life questionnaire consisting of five domains related to daily activities with 3-level answer possibilities. This will be completed by the patient (or via

For peer review only - http://bmjopen.bmj.com/site/about/guidelines.xhtml

parent/guardian proxy depending on the child's age) at baseline, 7 days, and 4 months post-randomisation.

Pain at dressing change

The level of pain experienced by the child at their first dressing change which occurs at 7 days will be assessed using a 3 point Pain Likert scale for children (based upon the Wong Baker Scale). This will be completed by the patient, or a parent/guardian proxy.

Cost-effectiveness

A health resource use questionnaire will be completed by the parent/guardian at 7 days and 4 months post-randomisation. This will collect information on hospital visits, dressing and antibiotic use and hospital readmission and repeat surgery.

Surgical site infection by 4 months

The presence of a SSI during the 4 month period post-randomisation will be assessed. In addition to the clinical assessment at 7 days, the patient's parent/guardian will be asked if the patient experienced any problems post-surgery. This will then be referred back to sites, where appropriate, to obtain confirmation from clinical notes and if necessary General Practitioner notes. This will capture any surgical site infections which occur after the usual expected timeframe in which infections would normally present.

Participant/parent satisfaction with nail healing

A patient assessment of the nail appearance (3-point Likert scale for children) will be used to measure patient reported satisfaction with the healing of the nail at 4 months post-randomisation. If the child cannot complete this score, a Visual

For peer review only

Analogue Scale (VAS) in the form of a measured line with a continuous scale (from 0 to 100) anchored by two verbal descriptors for each extreme symptom will be used as a patient proxy for measuring satisfaction with nail healing.

Study population

Inclusion criteria

- Male or female, aged below 16 years old at the time of presentation to the participating hospital.
- Nail bed injury occurring within 48 hours of presentation at trial centre believed to require surgical repair by the surgical team. This includes sharp lacerations, stellate lacerations, crush and avulsion injuries of the nail bed, injuries involving the sterile and/or germinal matrix, nail bed injuries with an associated pulp laceration and/or with an associated 'tuft' fracture of the distal phalanx.
- Patients whose parent or legal guardian consent to their inclusion in the trial and are willing to complete follow up, including photographs.
- Sufficient understanding of the child and parent/guardian participant information sheets as deemed by recruiting team at local sites.
- Single digit nail bed injury.

Exclusion criteria

The participant will not enter the trial if any of the following apply.

- Patients present with an infected nail bed injury.
- Patients have an underlying nail disease or deformity in the injured or contralateral finger prior to the injury.

For peer review only - http://bmjopen.bmj.com/site/about/guidelines.xhtml

- Patients have an associated distal phalanx fracture, requiring fixation with a Kirschner wire.
- Patients with an amputation of the distal fingertip including all or part of the nail bed.
- Patients with loss of part or all of the nail bed, requiring a nail bed graft or flap reconstruction.
- Previous NINJA trial participants.
- Patients with nail bed injuries to more than one digit.

Recruitment and consent

Trial participants will be prospectively recruited from the participating hospitals. Initial assessment will take place in the Accident and Emergency/Minor Injuries department or paediatric ward. The clinical team will identify any potential participants and refer on to the research team for further information. The research team will obtain informed consent. Screening logs will be maintained at each site. Reasons for non-participation and/or ineligibility will be documented.

Parents/guardians will be given an information sheet and have the trial explained to them by the researcher. Children will also be provided with age appropriate information in order to include them in the consent process. Consent for medical photography will be included as part of the consent process in order for the research team to analyse participant submitted photographs, and agreement to return follow up questionnaires and submit a photograph at a minimum of 4 months post-surgery will be part of the inclusion criteria.

For peer review only - http://bmjopen.bmj.com/site/about/guidelines.xhtml

Data collection

The baseline assessment will be on the day of the operation, before randomisation but after consent to participation. Participant demographics will be recorded on the Case Report Form (CRF) when the assessing surgeon on admission in the Emergency Department or the paediatric ward surgically reviews the participant. Follow up assessments will involve a clinical appointment around 7 days post operation and a participant reported questionnaire, sent via text, email or post, at around 7 days post operation and 4 months (Table 1 and Figure 1).

Randomisation and blinding

A web-based randomisation system will be provided by the Oxford Clinical Trials Research Unit (OCTRU). The allocations will be computer generated with a 1:1 ratio and stratified by site using random permuted blocks of varying size within stratum. Randomisation will take place when the participant is in the anaesthetic room just prior to surgery, or as close to the surgery time as possible by a good clinical practice (GCP) trained member of the team.

This is an open trial, since those delivering the care will not be blinded to the intervention the participant has been allocated to. This is because a replaced nail can take several weeks to loosen and fall off once a new nail has grown out and therefore the treatment received will be obvious within this timeframe. Therefore, the assessment of the photographs for cosmetic appearance at a minimum of 4 months will be done by independent assessors who can at that time point be blinded.

For peer review only - http://bmjopen.bmj.com/site/about/guidelines.xhtml

Operative assessment

At the time of surgery, the operating surgeon will classify the nail bed injury according to the system used and tested in the pilot (4).

Interventions

Nail bed repair

In both groups (nail plate replaced or nail plate discarded), the nail bed repair will be performed using 6/0 or 7/0 interrupted Vicryl Rapide (Johnson and Johnson Medical Ltd, Livingston, West Lothian, UK) or equivalent sutures. This is a pragmatic trial. The following decisions will be left to the discretion of the surgical team responsible for the participant, but recorded on the CRF:

- The type of anaesthetic used (general anaesthetic, local anaesthetic, or both)
- Perioperative antibiotics given, if any
- Type and duration of tourniquet used
- Type of surgical preparation solution and wash used
- Type of dressing applied. In practice this is usually a combination of a non-adherent dressing, absorbent layer and a top layer of fabric based dressing to keep the digit covered.

If the surgeon has to perform a procedure(s), which was part of the exclusion criteria, this will be recorded on the CRF. This is an extremely unusual event as the vast majority of these procedures (e.g. fracture fixation with a Kirschner wire, need for a composite graft or nail bed graft) are predictable pre-operatively. These participants will be analysed within the intention to treat analysis of the trial. In both groups the fingertip will be dressed with a non-

For peer review only - http://bmjopen.bmj.com/site/about/guidelines.xhtml

adherent dressing. The operating surgeon will add to the CRF the following data: the type of nail bed injury, whether the nail plate was replaced or discarded, whether a nail substitute was used, what, if any, antibiotics were given perioperatively and what postoperative antibiotic regime is planned.

Nail plate replaced

In the nail plate replaced group, the nail plate will be secured using a figure-of-eight vicryl rapide suture. If the nail plate cannot be replaced in a participant randomised to this group, for example if it is too badly damaged, a nail substitute of the operating surgeon’s choice will be used and recorded on the CRF.

Nail plate discarded

In the nail plate discarded group after the nail bed repair, the nail will not be replaced. It will be discarded appropriately instead. The washout, debridement, and suturing procedures will be the same as described for the first group.

Safety reporting

Data on adverse and serious adverse events will be recorded and their severity and frequency will be assessed. Standard HRA safety reporting measures will be adhered to. The OCTRU conducted a risk assessment prior to the trial starting. Issues raised have been addressed within the current approved protocol and procedures have been planned to monitor the on-going risks of the trial. A risk proportionate approach will be utilised within this trial. Central monitoring of trial procedures will be imbedded into the trial conduct and management, including instituting a trial steering committee (TSC) and data

For peer review only
For peer review only - http://bmjopen.bmj.com/site/about/guidelines.xhtml

monitoring committee (DMC). The TSC and DMC will agree their respective terms of reference. No formal statistical interim safety analysis has been planned for in the design, or are anticipated given the nature of the trial. The trial may be monitored, or audited in accordance with the current approved protocol, GCP, relevant regulations and standard operating procedures. The trial will be subject to audit according to OCTRU's Audit Programme.

End of trial

The end of trial is the date of the last follow up of the last participant.

Analysis

Statistical analysis

Principal analyses will be on an "as randomised" basis retaining participants in their randomised allocation groups irrespective of compliance to the allocation. A two sided 5% significance level will be adopted with associated 95% confidence intervals (CIs) whenever possible using appropriate summary measures (e.g. number of events and percentage for binary measures). The principal analyses will also be carried out on a complete case basis with sensitivity to missing data explored for the primary outcomes.

The number of participants

Sample size calculations are based on the co-primary outcomes of surgical site infection and cosmetic appearance at a minimum of 4 months, measured via the Oxford Finger Nail Appearance Score, the development of which was informed by the Zook nail classification scale (5) – a 0-5 ordinal summary score reflecting optimal or suboptimal appearance across the five classification

For peer review only - http://bmjopen.bmj.com/site/about/guidelines.xhtml

domains. Pilot data from our NINJA-P trial (4) showed a substantial proportion of participants did not have nails with optimal appearance (approximately 35% had two or more suboptimal aspects of appearance, i.e. score of three or less). Based upon a clinically relevant difference of 15% more achieving the optimal appearance score of 5 (from 35 to 50% with a corresponding shift in the other score values) and using a two-sided significance level of 0.05, 332 (166 per group) are required to obtain 90% power based upon a Mann-Whitney U test. After allowance for 20% missing data, a total of 416 participants (208 in each group) are required. This calculation was carried out using an extended version of the Excel spreadsheet provided by Walters (6) to allow for a six point ordinal outcome. Based upon a lower overall level and a smaller difference in the proportion with a surgical site infection than the one observed in the Miranda (2) observational study (8 vs 1%), this sample size is also sufficient for 90% power at the two-sided 5% significance level. This latter calculation was carried out in Stata 14 using the power twoprop command.

Analysis of outcome measures

As multiple assessors will be reviewing each photograph using the Oxford Finger Nail Appearance Score, the median of the assessors' total scores will be used as the rating for each photo to account for any variability in scores. These will then be analysed using a Mann-Whitney U test (with a 95% CI for the median also calculated). A secondary more complex ordinal regression model will also be used to estimate the difference across the ordinal scale and allow subgroup analyses.

For peer review only - http://bmjopen.bmj.com/site/about/guidelines.xhtml

Surgical site infection will be compared using logistic regression adjusted for site. If the number of events is too low for adjustment, univariate logistic regression will be carried-out. Pre-specified subgroup analysis will be carried out according to preoperative antibiotic use using a treatment-by-subgroup interaction extending the aforementioned regression models for the co-primary outcomes. Secondary outcomes will be analysed using generalised linear models as appropriate. Further details of the planned statistical analyses will be specified in a Statistical Analysis Plan, which will be finalised prior to the un-blinding of data to NINJA investigators. Available data will be used up to the point of withdrawal whenever possible.

Economic analysis

A within-trial cost-utility analysis comparing nail replacement with nail discarding will be conducted from the UK NHS and Personal Social Services perspective in the base case (or primary) analysis (7).

Resource use for the surgery will be recorded by the research team in the CRF while data for the economic evaluation will be collected from the trial questionnaires given to participants at around 7 days and at a minimum of 4 months after randomisation. Unit cost of this resource use will be sourced from the latest NHS Supply Chain Catalogue, NHS Reference Cost and British National Formulary. Where appropriate, the cost of health resource use per patient will be computed by multiplying the frequency of health resource utilisation with the unit cost of each resource item.

Health-related quality of life (HRQoL) will be estimated using the EQ-5D-Y questionnaire at baseline, at around 7 days and at a minimum of 4 months. The

For peer review only - http://bmjopen.bmj.com/site/about/guidelines.xhtml

EQ-5D-Y user guide instructions will be followed so that children are given age-appropriate questionnaires to answer (8).

A cost-utility analysis (excluding the participants below the age of 2) will present outputs of the analyses in terms of incremental cost-effectiveness ratio (ICER) where the NICE cost-effectiveness threshold of £20,000-£30,000 per additional QALY will be applied. Given the methodological limitations surrounding preference-based outcomes measurement in young children, a cost-effectiveness analysis will also be conducted (for the entire sample) where outputs will be expressed in terms of incremental cost per surgical site infections prevented.

If data are missing at random, multiple imputation analysis will be performed to avoid bias associated with the complete case analysis. We assume no outcome differences in terms of QoL, pain and complications beyond the trial period, therefore no longer-time perspective will be considered.

Sensitivity analysis such as extending the study perspective to societal perspective and assessing the impact of missing data on the ICERs will be performed. In order to assess sampling uncertainty on the ICERs and varying willingness-to-pay levels for an additional QALY, probabilistic sensitivity analysis (PSA) will be performed. Results from the PSA will be presented in cost-effectiveness acceptability curves, which will be generated via non-parametric bootstrapping.

Patient and public involvement

To inform study design 30 parents of children with nail bed injuries were surveyed. The survey identified normal regrowth of the nail, infection and long-

For peer review only - http://bmjopen.bmj.com/site/about/guidelines.xhtml

term appearance as the most common parental concerns following nail bed surgery (1). Subsequently a focus group and youth group refined follow-up methods, types of study material, as well as which outcomes were important. To ensure on-going patient and public involvement, a patient/carer representative is actively involved in general trial management. In addition, further independent patient/carer representatives are members of the steering committee.

Ethics and dissemination

This trial is conducted in accordance with the principles of the Declaration of Helsinki, with relevant regulations and with Good Clinical Practice. It has been approved by the South Central Research Ethics Committee (Berkshire-B, 04/06/2019, ref: 18/SC/0024). The participants in this trial are children and consent for them to take part will need to be obtained from their parent or legal guardian by a GCP trained research team member. If a child wishes not to take part in the trial, this will be respected. Personal information will be handled confidentially in line with GDPR regulations. Any publication arising out of the trial will follow the NIHR publication policy.

Figure 1 **Trial flow chart**

For peer review only

For peer review only - http://bmjopen.bmj.com/site/about/guidelines.xhtml

REFERENCES

1. Sierakowski A, Gardiner MD, Jain A, Greig AV, Nail bed INJury Analysis (NINJA) Collaborative Group. Surgical treatment of paediatric nail bed injuries in the United Kingdom: Surgeon and patient priorities for future research. J Plast Reconstr Aesthet Surg. 2016 Feb;69(2):286–8.

2. Miranda BH, Vokshi I, Milroy CJ. Pediatric Nailbed Repair Study. Plast Reconstr Surg. 2012 Feb;129(2):394e–396e.

3. Capstick R, Giele H. Interventions for treating fingertip entrapment injuries in children. Capstick R, editor. Cochrane database of systematic reviews (Online). Chichester, UK: John Wiley & Sons, Ltd; 2014 Apr 30;(4):CD009808.

4. Greig A, Gardiner MD, Sierakowski A, Zweifel CJ, Pinder RM, Furniss D, et al. Randomized feasibility trial of replacing or discarding the nail plate after nail-bed repair in children. Br J Surg. 2017 Nov;104(12):1634–9.

5. Zook EG, Guy RJ, Russell RC. A study of nail bed injuries: causes, treatment, and prognosis. YJHSU. 1984 Mar;9(2):247–52.

6. Walters SJ. Sample size and power estimation for studies with health related quality of life outcomes: a comparison of four methods using the SF-36. Health Qual Life Outcomes. 2004 May 25;2:26.

7. National Institute for Health and Care Excellence. Guide to the Methods of Technology Appraisal 2013. London: National Institute for Health and Care Excellence (NICE); 2013.

For peer review only - http://bmjopen.bmj.com/site/about/guidelines.xhtml

8. Van Reenen M, Janssen B, Oppe M, Group SKRE, 2014. EQ-5D-Y user guide: basic information on how to use the EQ-5D-Y instrument.

For peer review only

Table 1. Objectives and outcome measures

**Objectives**	**Outcome Measures**	**Timepoint(s) of evaluation of this outcome measure (if applicable)**
**Primary Objectives**<br>To assess the effects of replacing or discarding the fingernail by comparing the risk of infection and cosmetic appearance.	• Incidence of surgical site infection (clinical assessment around 7 days and participant or parent/guardian reported with clinical notes at a minimum of 4 months if information is relevant to earlier time-period).<br>• Oxford Finger Nail Appearance Score assessing nail appearance at a minimum of 4 months, considering 5 domains (shape, adherence, eponychium, surface quality and presence of split).	• 7 days<br>• At a minimum of 4 months
**Secondary Objectives**<br>To assess whether there is a difference in participant/parent & guardian reported health-related quality of life according to whether the nail is replaced or discarded.	• EuroQol EQ-5D-Y, and proxy completed by the child/parent or guardian according to the age of the participant	• Baseline, 7 days and a minimum of 4 months
To assess whether there is a difference in participant or parent/guardian -reported pain experienced between replacing and discarding the nail.	• The level of pain experienced by the child at their first dressing change assessed by the child or parent/guardian (3 point Likert scale for children)	• 7 days
To conduct a parallel within-trial economic analysis to assess the cost effectiveness (including resource use) of replacing versus discarding the nail	• Healthcare resource utilisation such as increased hospital visits, dressing and antibiotic use and in some cases hospital readmission and repeat surgery.	• 7 days and 4 months
To assess if any surgical site infection has occurred within the 4 months since surgery.	• Participant or parent/guardian reported incidence of infection with clinical notes confirmation.	• At a minimum of 4 months
To assess participant/parent satisfaction with nail healing	• Child or parent/guardian satisfaction with nail healing (3 point Likert scale for the	• At a minimum of 4 months

For peer review only - http://bmjopen.bmj.com/site/about/guidelines.xhtml

	children and a VAS score for the parents/guardians).	

For peer review only

For peer review only - http://bmjopen.bmj.com/site/about/guidelines.xhtml

For peer review only

For peer review only - http://bmjopen.bmj.com/site/about/guidelines.xhtml

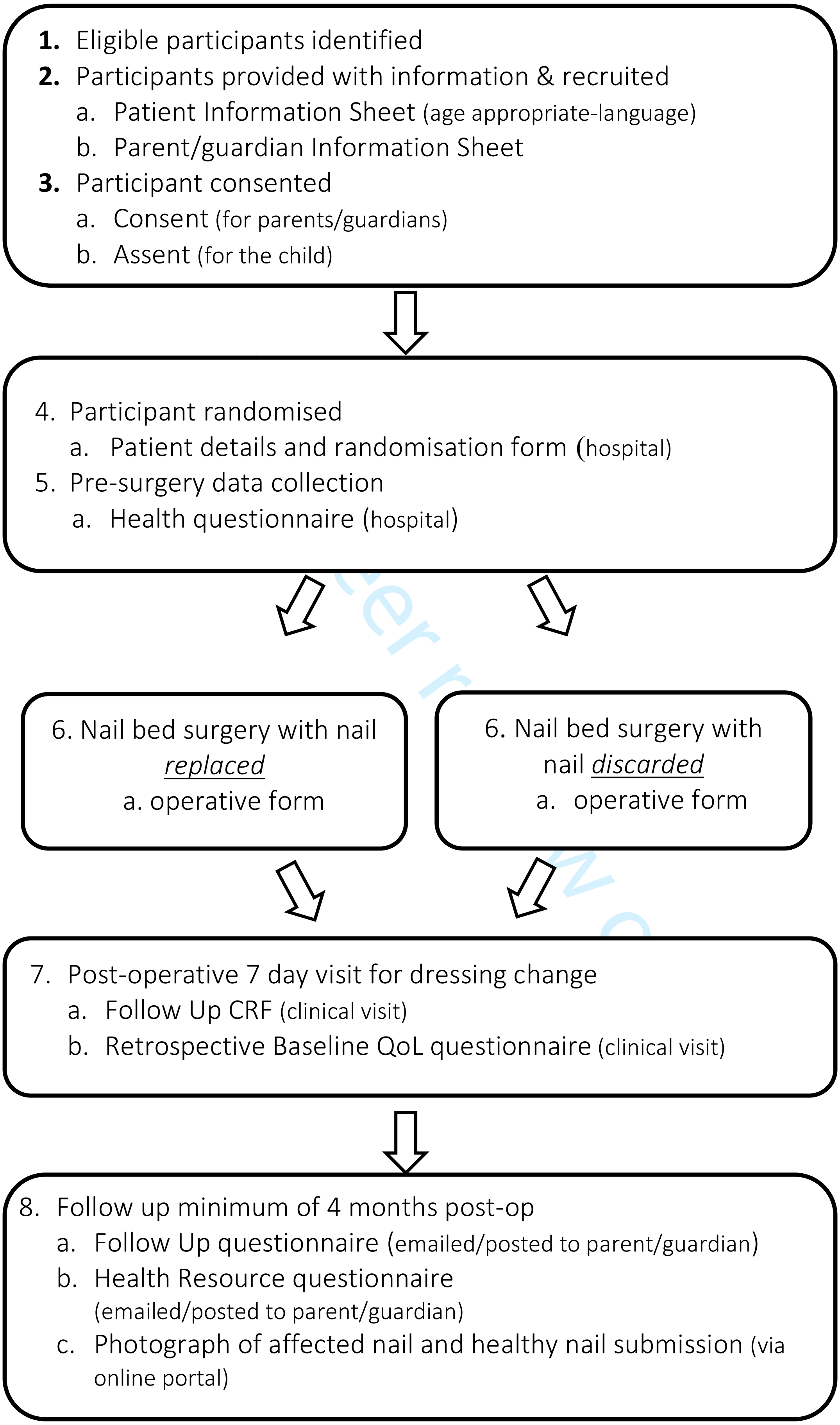

For peer review only - http://bmjopen.bmj.com/site/about/guidelines.xhtml

Reporting checklist for protocol of a clinical trial.

Based on the SPIRIT guidelines.

Instructions to authors

Complete this checklist by entering the page numbers from your manuscript where readers will find each of the items listed below.

Your article may not currently address all the items on the checklist. Please modify your text to include the missing information. If you are certain that an item does not apply, please write "n/a" and provide a short explanation.

Upload your completed checklist as an extra file when you submit to a journal.

In your methods section, say that you used the SPIRITreporting guidelines, and cite them as:

Chan A-W, Tetzlaff JM, Altman DG, Laupacis A, Gøtzsche PC, Krleža-Jerić K, Hróbjartsson A, Mann H, Dickersin K, Berlin J, Doré C, Parulekar W, Summerskill W, Groves T, Schulz K, Sox H, Rockhold FW, Rennie D, Moher D. SPIRIT 2013 Statement: Defining standard protocol items for clinical trials. Ann Intern Med. 2013;158(3):200-207

		Reporting Item	Page Number
**Administrative information**			
Title	#1	Descriptive title identifying the study design, population, interventions, and, if applicable, trial acronym	1
Trial registration	#2a	Trial identifier and registry name. If not yet registered, name of intended registry	5
Trial registration: data set	#2b	All items from the World Health Organization Trial Registration Data Set	5
Protocol version	#3	Date and version identifier	5
Funding	#4	Sources and types of financial, material, and other support	3
Roles and responsibilities: contributorship	#5a	Names, affiliations, and roles of protocol contributors	2

For peer review only

Roles and responsibilities: sponsor contact information	#5b	Name and contact information for the trial sponsor	2
Roles and responsibilities: sponsor and funder	#5c	Role of study sponsor and funders, if any, in study design; collection, management, analysis, and interpretation of data; writing of the report; and the decision to submit the report for publication, including whether they will have ultimate authority over any of these activities	2
Roles and responsibilities: committees	#5d	Composition, roles, and responsibilities of the coordinating centre, steering committee, endpoint adjudication committee, data management team, and other individuals or groups overseeing the trial, if applicable (see Item 21a for data monitoring committee)	2
**Introduction**			
Background and rationale	#6a	Description of research question and justification for undertaking the trial, including summary of relevant studies (published and unpublished) examining benefits and harms for each intervention	6
Background and rationale: choice of comparators	#6b	Explanation for choice of comparators	6
Objectives	#7	Specific objectives or hypotheses	7
Trial design	#8	Description of trial design including type of trial (eg, parallel group, crossover, factorial, single group), allocation ratio, and framework (eg, superiority, equivalence, non-inferiority, exploratory)	8
**Methods: Participants, interventions, and outcomes**			
Study setting	#9	Description of study settings (eg, community clinic, academic hospital) and list of countries where data will be collected. Reference to where list of study sites can be obtained	8
Eligibility criteria	#10	Inclusion and exclusion criteria for participants. If applicable, eligibility criteria for study centres and individuals who will	11

For peer review only

		perform the interventions (eg, surgeons, psychotherapists)	
Interventions: description	#11a	Interventions for each group with sufficient detail to allow replication, including how and when they will be administered	8
Interventions: modifications	#11b	Criteria for discontinuing or modifying allocated interventions for a given trial participant (eg, drug dose change in response to harms, participant request, or improving / worsening disease)	8
Interventions: adherance	#11c	Strategies to improve adherence to intervention protocols, and any procedures for monitoring adherence (eg, drug tablet return; laboratory tests)	12
Interventions: concomitant care	#11d	Relevant concomitant care and interventions that are permitted or prohibited during the trial	12
Outcomes	#12	Primary, secondary, and other outcomes, including the specific measurement variable (eg, systolic blood pressure), analysis metric (eg, change from baseline, final value, time to event), method of aggregation (eg, median, proportion), and time point for each outcome. Explanation of the clinical relevance of chosen efficacy and harm outcomes is strongly recommended	9
Participant timeline	#13	Time schedule of enrolment, interventions (including any run-ins and washouts), assessments, and visits for participants. A schematic diagram is highly recommended (see Figure)	8
Sample size	#14	Estimated number of participants needed to achieve study objectives and how it was determined, including clinical and statistical assumptions supporting any sample size calculations	17
Recruitment	#15	Strategies for achieving adequate participant enrolment to reach target sample size	17
**Methods: Assignment of interventions (for controlled trials)**			
Allocation: sequence generation	#16a	Method of generating the allocation sequence (eg, computer-generated random numbers), and list of any factors for stratification. To reduce predictability of a random sequence, details of any planned restriction (eg, blocking) should be provided in a separate document that is unavailable to those who enrol participants or assign interventions	13

For peer review only

Allocation concealment mechanism	#16b	Mechanism of implementing the allocation sequence (eg, central telephone; sequentially numbered, opaque, sealed envelopes), describing any steps to conceal the sequence until interventions are assigned	13
Allocation: implementation	#16c	Who will generate the allocation sequence, who will enrol participants, and who will assign participants to interventions	13
Blinding (masking)	#17a	Who will be blinded after assignment to interventions (eg, trial participants, care providers, outcome assessors, data analysts), and how	13
Blinding (masking): emergency unblinding	#17b	If blinded, circumstances under which unblinding is permissible, and procedure for revealing a participant's allocated intervention during the trial	13
**Methods: Data collection, management, and analysis**			
Data collection plan	#18a	Plans for assessment and collection of outcome, baseline, and other trial data, including any related processes to promote data quality (eg, duplicate measurements, training of assessors) and a description of study instruments (eg, questionnaires, laboratory tests) along with their reliability and validity, if known. Reference to where data collection forms can be found, if not in the protocol	13
Data collection plan: retention	#18b	Plans to promote participant retention and complete follow-up, including list of any outcome data to be collected for participants who discontinue or deviate from intervention protocols	13
Data management	#19	Plans for data entry, coding, security, and storage, including any related processes to promote data quality (eg, double data entry; range checks for data values). Reference to where details of data management procedures can be found, if not in the protocol	12
Statistics: outcomes	#20a	Statistical methods for analysing primary and secondary outcomes. Reference to where other details of the statistical analysis plan can be found, if not in the protocol	16
Statistics: additional analyses	#20b	Methods for any additional analyses (eg, subgroup and adjusted analyses)	16

For peer review only

Statistics: analysis population and missing data	#20c	Definition of analysis population relating to protocol non-adherence (eg, as randomised analysis), and any statistical methods to handle missing data (eg, multiple imputation)	16
**Methods: Monitoring**			
Data monitoring: formal committee	#21a	Composition of data monitoring committee (DMC); summary of its role and reporting structure; statement of whether it is independent from the sponsor and competing interests; and reference to where further details about its charter can be found, if not in the protocol. Alternatively, an explanation of why a DMC is not needed	15
Data monitoring: interim analysis	#21b	Description of any interim analyses and stopping guidelines, including who will have access to these interim results and make the final decision to terminate the trial	15
Harms	#22	Plans for collecting, assessing, reporting, and managing solicited and spontaneously reported adverse events and other unintended effects of trial interventions or trial conduct	15
Auditing	#23	Frequency and procedures for auditing trial conduct, if any, and whether the process will be independent from investigators and the sponsor	15
**Ethics and dissemination**			
Research ethics approval	#24	Plans for seeking research ethics committee / institutional review board (REC / IRB) approval	3
Protocol amendments	#25	Plans for communicating important protocol modifications (eg, changes to eligibility criteria, outcomes, analyses) to relevant parties (eg, investigators, REC / IRBs, trial participants, trial registries, journals, regulators)	15
Consent or assent	#26a	Who will obtain informed consent or assent from potential trial participants or authorised surrogates, and how (see Item 32)	12
Consent or assent: ancillary studies	#26b	Additional consent provisions for collection and use of participant data and biological specimens in ancillary studies, if applicable	12
Confidentiality	#27	How personal information about potential and enrolled participants will be collected, shared, and maintained in order to protect confidentiality before, during, and after the trial	20

For peer review only

Declaration of interests	#28	Financial and other competing interests for principal investigators for the overall trial and each study site	3
Data access	#29	Statement of who will have access to the final trial dataset, and disclosure of contractual agreements that limit such access for investigators	16
Ancillary and post trial care	#30	Provisions, if any, for ancillary and post-trial care, and for compensation to those who suffer harm from trial participation	n/a
Dissemination policy: trial results	#31a	Plans for investigators and sponsor to communicate trial results to participants, healthcare professionals, the public, and other relevant groups (eg, via publication, reporting in results databases, or other data sharing arrangements), including any publication restrictions	20
Dissemination policy: authorship	#31b	Authorship eligibility guidelines and any intended use of professional writers	20
Dissemination policy: reproducible research	#31c	Plans, if any, for granting public access to the full protocol, participant-level dataset, and statistical code	20
**Appendices**			
Informed consent materials	#32	Model consent form and other related documentation given to participants and authorised surrogates	n/a
Biological specimens	#33	Plans for collection, laboratory evaluation, and storage of biological specimens for genetic or molecular analysis in the current trial and for future use in ancillary studies, if applicable	n/a

The SPIRIT checklist is distributed under the terms of the Creative Commons Attribution License CC-BY-ND 3.0. This checklist was completed on 08. May 2019 using https://www.goodreports.org/, a tool made by the EQUATOR Network in collaboration with Penelope.ai

For peer review only
